# Facile Preparation of Nanostructured, Superhydrophobic Filter Paper for Efficient Water/Oil Separation

**DOI:** 10.1371/journal.pone.0151439

**Published:** 2016-03-16

**Authors:** Jianhua Wang, Jessica X. H. Wong, Honoria Kwok, Xiaochun Li, Hua-Zhong Yu

**Affiliations:** 1 Key Laboratory of Advanced Transducers and Intelligent Control Systems (Ministry of Education and Shanxi Province), College of Physics and Optoelectronics, Taiyuan University of Technology, Shanxi, 030024, P.R. China; 2 Department of Chemistry, Simon Fraser University, Burnaby, British Columbia, V5A 1S6, Canada; Brandeis University, UNITED STATES

## Abstract

In this paper, we present a facile and cost-effective method to obtain superhydrophobic filter paper and demonstrate its application for efficient water/oil separation. By coupling structurally distinct organosilane precursors (e.g., octadecyltrichlorosilane and methyltrichlorosilane) to paper fibers under controlled reaction conditions, we have formulated a simple, inexpensive, and efficient protocol to achieve a desirable superhydrophobic and superoleophilic surface on conventional filter paper. The silanized superhydrophobic filter paper showed nanostructured morphology and demonstrated great separation efficiency (up to 99.4%) for water/oil mixtures. The modified filter paper is stable in both aqueous solutions and organic solvents, and can be reused multiple times. The present study shows that our newly developed binary silanization is a promising method of modifying cellulose-based materials for practical applications, in particular the treatment of industrial waste water and ecosystem recovery.

## Introduction

In recent years, pollution caused by the leakage of oil and other chemical reagents into water occurs so frequently, that this has become a serious global problem. Since contaminated water usually contains harmful or toxic organic materials and therefore threatens human health, effective measures should be taken to remove the oily contaminants [1]. For this reason, many analytical methods have been explored, including physical extraction [2] and biochemical degradation [3], though the latter protocol may result in secondary pollution, similar to a combustion process. In comparison, physical extraction of oil contaminants possesses a distinct advantage, and therefore, newly developed superhydrophobic and superoleophilic materials have drawn broad attention because of their selective absorption of oil and repellence of water. In addition to exploring traditional absorbent materials for oil/water separation [4–11], various artificial superhydrophobic materials have been prepared, such as hydrophobic aerogels [9], nanoporous polydivinylbenzene [10], and nanostructured hydrogel-coated metal mesh [1]. Thus far, varying degrees of success have been achieved with these new materials; there are still inevitable limitations such as high cost, low separation efficiency, and poor recyclability. Among various challenges, they are not easy to be properly disposed of after use. Novel materials that are cost-effective, reusable, and easy-to-dispose are still highly sought after for efficient oil/water separation.

Cellulose is the main raw material of paper and cotton fabrics; it is not only abundant in nature, but also light in weight, stable, and easily disposed of. More importantly, cellulose-based materials (e.g., filter paper) can be chemically modified for various new applications [12–19]. Typically, a superhydrophobic and superoleophilic surface can be achieved by dipping, spin-coating or spraying filter paper with a solution, suspension, or emulsion of hydrophobic nanoparticles and pigments [13–16]. For example, Li et al. recently adopted a simple spray-coating process using octadecyltrichlorosilane (OTS)-modified SiO_2_ nanoparticles (~ 50 nm in diameter) in an ethanol suspension to create a superhydrophobic and superoleophilic coating on paper [17]. Independently, Zhang et al. used rather large OTS-modified silica particles (~ 243 nm) in a THF solution containing polystyrene to prepare a superhydrophobic coating on filter paper [18]. Up to date, very limited studies have been carried out for using modified cellulose-based materials for oil/water separation [18–23]. Meanwhile, other traditional materials (*vide supra*) have been widely tested for oil/water separation with satisfactory separation efficiencies [17]; they are mostly stable and reusable, but have undesirable properties such as high costs, disposal difficulties, and complex fabrication procedures (e.g., polymers must be cross-linked or grafted [1,7], or multiple modification steps are required [5,6]).

In this paper, we present a facile and cost-effective method to obtain a hydrophobic and oleophilic surface on filter paper and demonstrate its application for efficient oil/water separation. Our approach stems from the pioneering studies of coating paper with hydrophobic nanoparticles [13, 17–19] and conventional salinization reactions to modify hydroxylated surfaces with single-component long-chain alkylsilanes [24–27], but it is different in terms of the selection of reaction precursors and control of experimental conditions. Particularly, we have explored the application of a binary octadecyltrichlorosilane (OTS) / methyltrichlorosilane (MTS) solution in n-hexane to directly treat filter paper, by which we were able to achieve a superhydrophobic surface within 10 min. The nanostructured morphology of the modified filter paper was examined and its separation efficiency for different oil/water mixtures (e.g., diesel or gasoline mixed with water) investigated in detail. In addition, the acid/alkali stability of the superhydrophobic filter paper and its resistance to different organic solvents was thoroughly tested.

## Results and Discussion

### Fabrication of superhydrophobic filter paper by binary silanization

**Fig 1:**
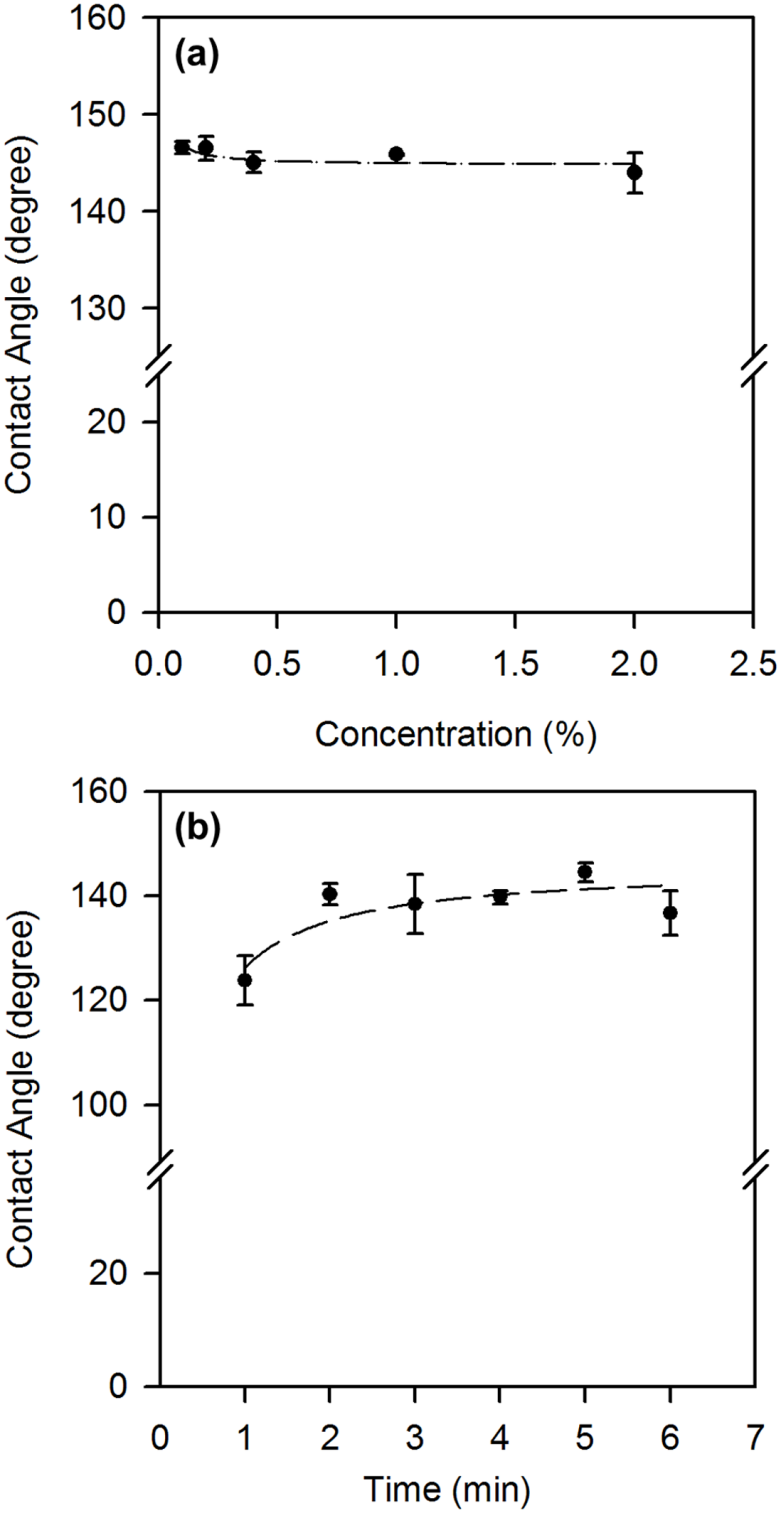
Water contact angle as a function of (a) OTS concentration in n-hexane at a fixed reaction time of 8 min and (b) reaction time of OTS on filter paper using a concentration of 0.1% OTS.

Different from typical coating protocols with hydrophobic particles [13, 17–19], we formulated a convenient method for filter paper modification by simply immersing a piece of filter paper into a binary solution of OTS and MTS at ambient temperature, followed by oven drying. The reactions between organosilanes with hydroxylated surfaces other than cellulose fibers have been studied in the past [24–27]. Typically long chain alkyltrichlorosilanes react with the hydroxyl groups on surface in the presence of trace amounts of water, and then cross-link with neighboring silane molecules to form a densely packed monolayer; nonetheless the short silanes tend to form 3D networks [24–27]. The reaction between a mixture of short and long alkyltrichlorosilanes and a hydroxylated surface has not been studied in the past; we started our investigation with the optimization of the reaction conditions, namely the OTS/MTS ratio in n-hexane and the reaction time. The optimization of the OTS/MTS ratio was achieved by testing a series of ratios (9:1 to1:9, total silane concentration of 0.2% v/v), first on Whatman^™^ No. 3, for a set time period (data not shown). Of the tested ratios, a 3:7 OTS:MTS ratio was found to have the most ideal wetting properties (highest water contact angles); this ratio was then used to prepare modified filter papers with different reaction times. Depending on the type of filter paper used, the water contact angles on the filter papers upon binary silanization were from 141±3° to 153±2° (Table 1), which are much higher than that of standard printing paper (124±4°). More importantly, it only took less than 10 min to achieve a reproducible, stable superhydrophic paper. For the purpose of oil/water separation, we will focus our investigation on Whatman^™^ No. 1 filter paper, which has a high flow rate (water) of 57 mL/min and is low cost, but adequate performance in separations [28]. Particularly for this type of filter paper, we found that the optimal concentration of OTS was 0.1% (v/v) (without the addition of MTS) (Fig 1a); the water contact angle for the modified surface was 145±3°, and a reaction time of 5 min was determined to be sufficient (Fig 1b).

**Table 1:** Modification of different types of filter paper and standard printing paper. The pore sizes and flow rates [28] are listed for comparison.

Paper type	Pore size / μm	Water flow rate / mL/min	Water contact angle /degree	Application
Whatman^™^, No. 1	11	57	142±4	Traditional qualitative analytical separations
Whatman^™^, No. 3	6	28	153±2	Quantitative analytical separations: double the thickness of grade 1, for the retention of finer particles
Whatman^™^, No. 541	22	359	141±3	Quantitative analysis: reduces ash to an extremely low level
Printing paper	n/a	n/a	124±4	Office general use

Untreated filter paper is both hydrophilic and oleophilic, i.e., both water and oil spread and absorb quickly into the paper, while for modified hydrophobic filter paper (with a water contact angle of 145±3°), however, this is not the case as water does not penetrate through the hydrophobic layer into the paper. The water droplets instead form a bead on the surface, although even at these high contact angles, the water droplets don’t roll off easily, i.e., these samples do not have low sliding angles, similar to other “sticky” superhydrophobic surfaces reported [29–31]. It should be noted that the adherence of the water droplets to the surface does not hinder the absorption of gasoline and other organic solvents through the paper, thus ensuring the oil/water separating ability (*vide infra*).

**Fig 2:**
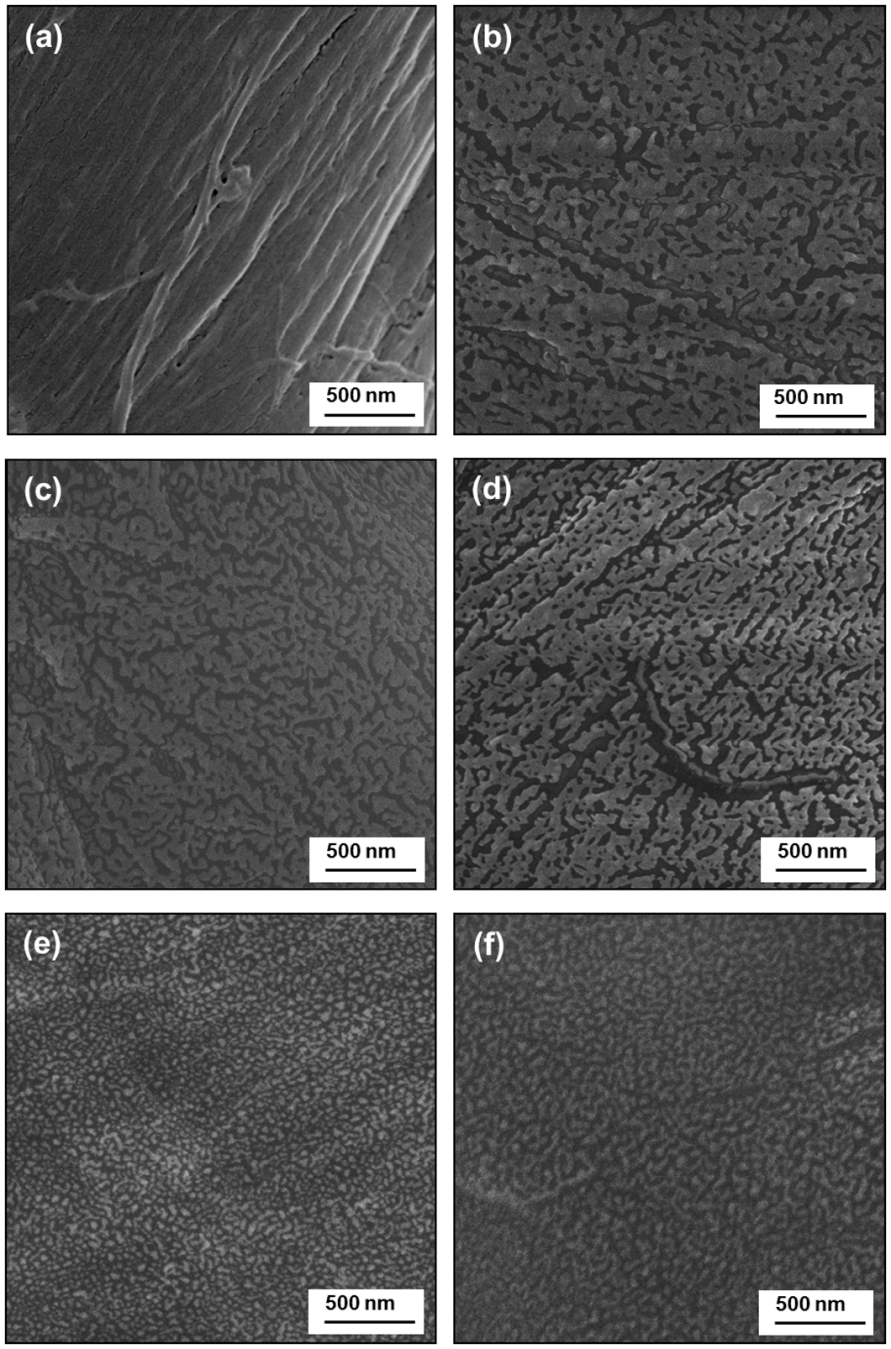
SEM images showing the surface morphology of untreated filter paper (a) and those modified with 0.2% OTS, 7:3 OTS/MTS with a total concentration of 0.2% (c), 0.2% MTS (d), 0.4% OTS (e), and 1% OTS (f), respectively.

Scanning electron microscopy (SEM) images of the filter papers were obtained to examine the surface morphology before and after the silanization treatment (Fig 2). As shown in Fig 2a, the untreated filter paper appears fibrous and smooth. After silanization (Fig 2b–2f), while the fibrous textures can still identified (e.g., the horizontal textures in Fig 2b), the fibers now appear rougher as the surface is covered with particulate nanostructures and these structures are present regardless of the reaction conditions (Fig 2b–2f). The micro-(fibers) and nanostructures are likely responsible for the superhydrophobic state, i.e., the ability to trap air pockets between the particulate nanostructures [32]. These particulates are believed to be the cross-linked networks produced by the hydrolysis and condensation of the silanes. The SEM images of filter paper modified with different silanes and their mixture at the same (total) silane concentration showed no significant differences (Fig 2b–2d) despite the observed variations in the water contact angles. In fact, the greatest dependence in the morphology resulted from the different silane concentrations; as the concentration of OTS was increased to 0.4%, the particulate nanostructures became more distinct, i.e., instead of networked nanostructures isolated particles are clearly visible (Fig 2e and 2f). We note that in-depth morphological studies and their relationship with the silanization condition are warranted and currently underway in our laboratory; this report will focus on the application aspect of modified filter paper for oil/water separation.

### Separation of oil/water mixtures with silanized filter paper

**Fig 3:**
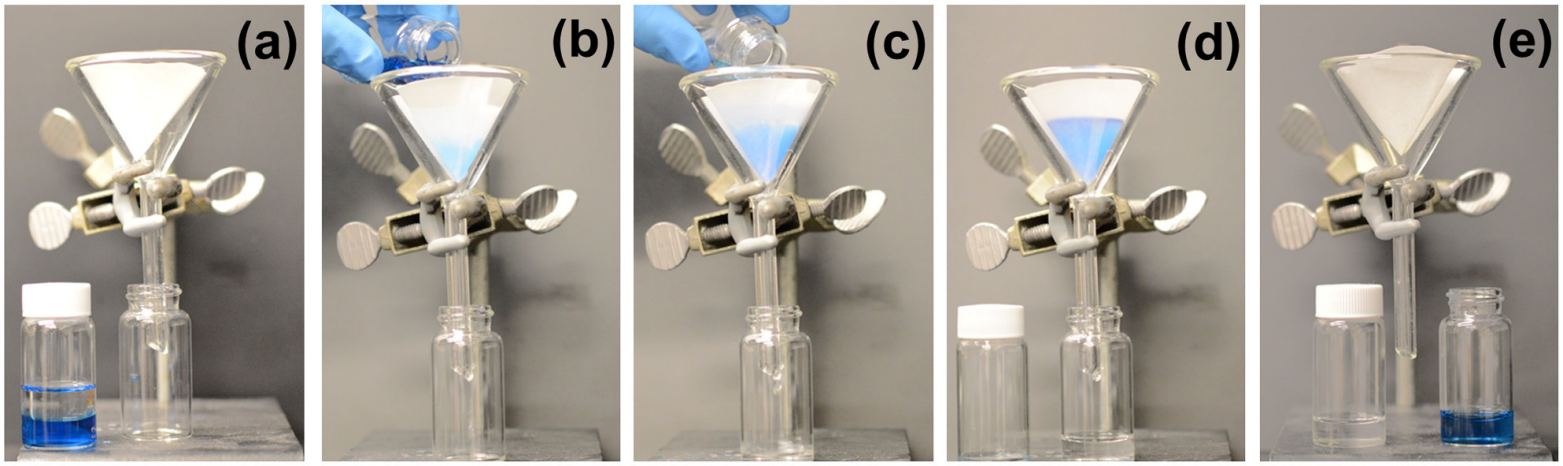
Separation of an oil/water mixture. The water layer has been dyed blue to allow for easier visualization. The mixture is poured onto the silanized filter paper fitted on a glass funnel. As the colorless oil layer permeates the filter paper (b-d), the water layer (blue) remains. The water layer is finally collected into a new vial and showed beside the oil sample (e).

With an average water contact angle of 145±3°, the modified filter paper (Whatman^™^, grade 1) is not as “superhydrophobic” as other materials previously developed for the oil/water separation [1, 5, 6, 14, 16, 19, 24, 33]; our experimental results however, demonstrate that such a nanostructured, (pseudo) superhydrophobic filter paper can selectively repel water and allow oil to pass through readily, as shown in Fig 3. In this experiment, deionized water, dyed blue, was mixed with hexane (Fig 3a) and then poured onto a modified filter paper fitted on a glass funnel. The mixture was allowed to be filtered through (Fig 3b–3d); only the organic layer penetrates the filter paper and is collected into the vial below, leaving the water layer atop the filter paper.

**Fig 4:**
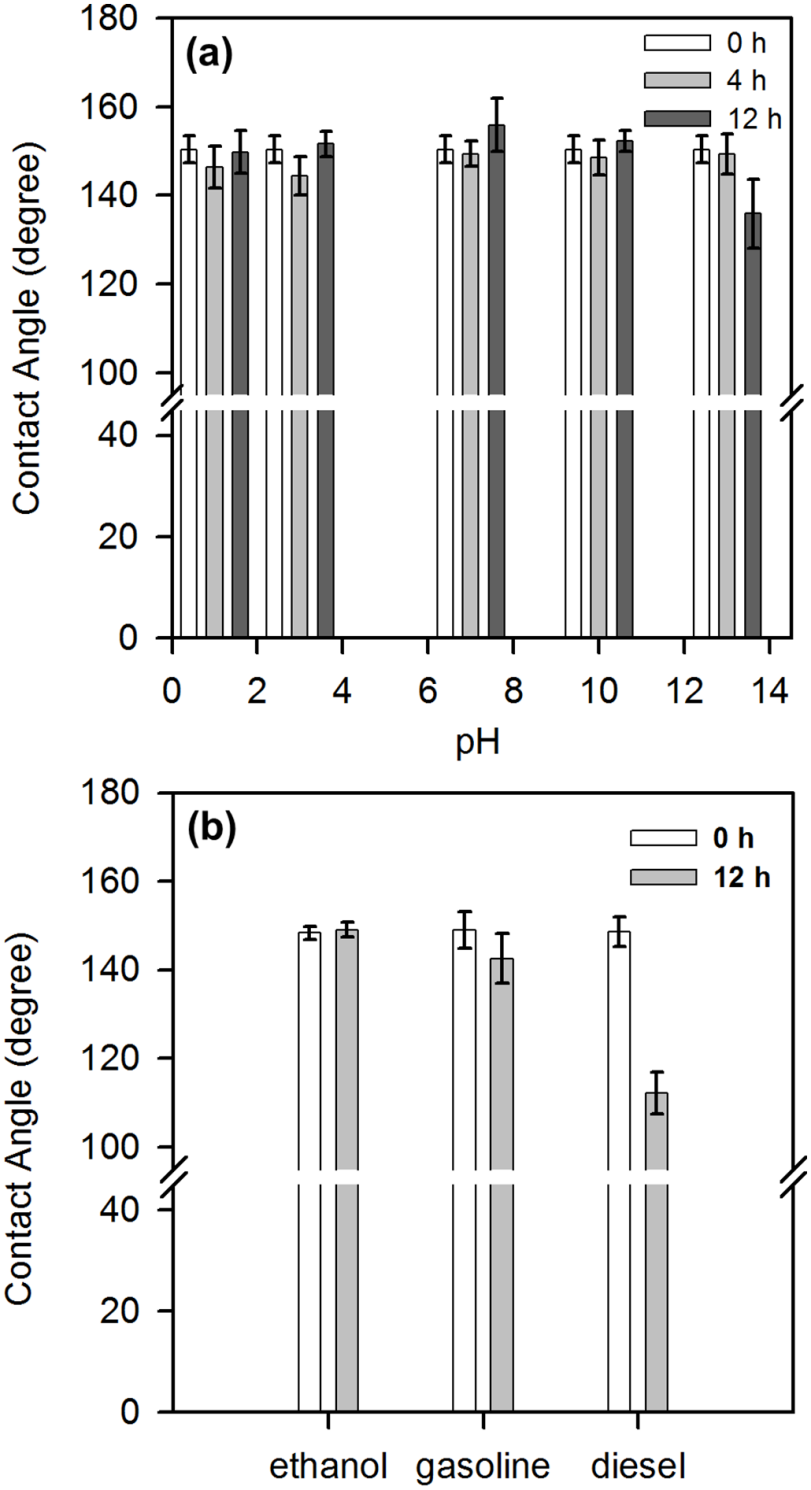
Relationship between the water contact angle on modified, hydrophobic filter paper after immersion in (a) solutions of different pH for different periods of time; (b) different organic solutions for 12 h.

Before we studied the efficiency of the modified filter paper in separating oil/water mixtures, we investigated its stability in both acidic and basic conditions. As shown in Fig 4a, immersion in solutions at different pH only had a minimal impact on the water contact angles of the hydrophobic filter paper, even after 4 and 12 hours. Between pH 1 and 11, water contact angles were all greater than 140°, and the variation was smaller than 10°. Only at a high pH (13) and a prolonged immersion time (12 h) did the water contact angle decreased slightly (~135°). In addition, the silanized filter paper remained superhydrophobic upon immersion in organic solvents, with an insignificant decrease in its water contact angle. For ethanol, the variation in water contact angle was smaller than 3° and for gasoline it was less than 10° after 12 h (Fig 4b). For diesel, the change in water contact angles was somewhat larger (decreased to about 110°), but the filter paper still remained hydrophobic. This result suggests that organic solvents have insignificant influence on the hydrophobicity of the modified filter paper.

**Fig 5:**
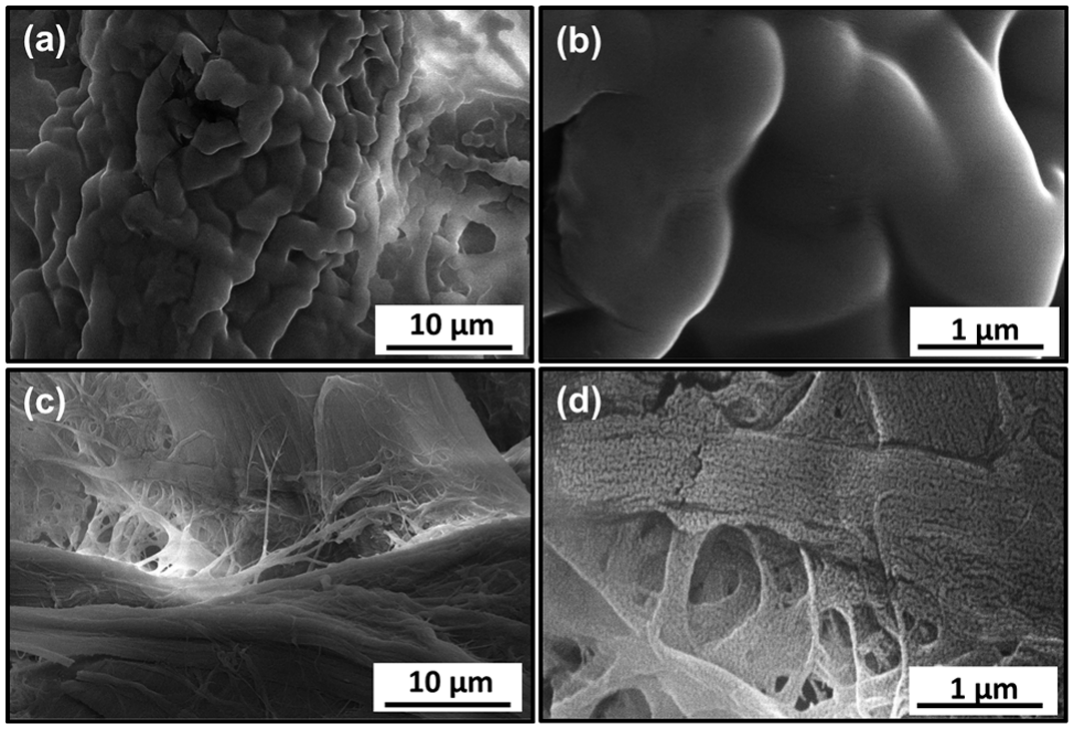
SEM images of the silanized filter paper after immersion in diesel (a, b) and gasoline (c, d) for 12 hours.

To correlate with potential morphological changes, we carried out SEM studies of the modified filter paper upon immersion in organic solvents. As shown in Fig 5, the microscale fiber structure is similar to the freshly modified filter paper. However, the nanoparticulate structures are no longer as distinguishable as before the immersion. In the case of filter paper that has come in contact with diesel (Fig 5a and 5b), it appears as though the particulate nanostructures have been covered by a layer of “soft materials”, possibly long-chain hydrocarbons or other contaminants in the fuel. Likewise, the particulate nanostructures on the modified filter paper that have been treated with gasoline are coated with a soft layer (possibly short-chain hydrocarbons), enlarging the particle size, but the nanoscale structures still remain. This may explain the difference between diesel and gasoline in terms of the decrease in water contact angles before and after immersion. As a result of coating the nanostructures, the “particles” on the diesel sample have become so large that they are no longer at the nanoscale, unable to trap air pockets to create a Cassie-Baxter state, instead retaining the hydrophobicity of the Wenzel state [34]. The gasoline immersion differs in that although the nanoparticles become slightly larger (Fig 5c and 5d), they remain at the nanoscale, such that the filter paper retains its superhydrophobicity. These results demonstrate that the as-prepared filter paper can be considered to have a good resistance to both acidic and alkaline solutions, as well as to some organic solvents, making it a suitable candidate for practical applications.

**Fig 6:**
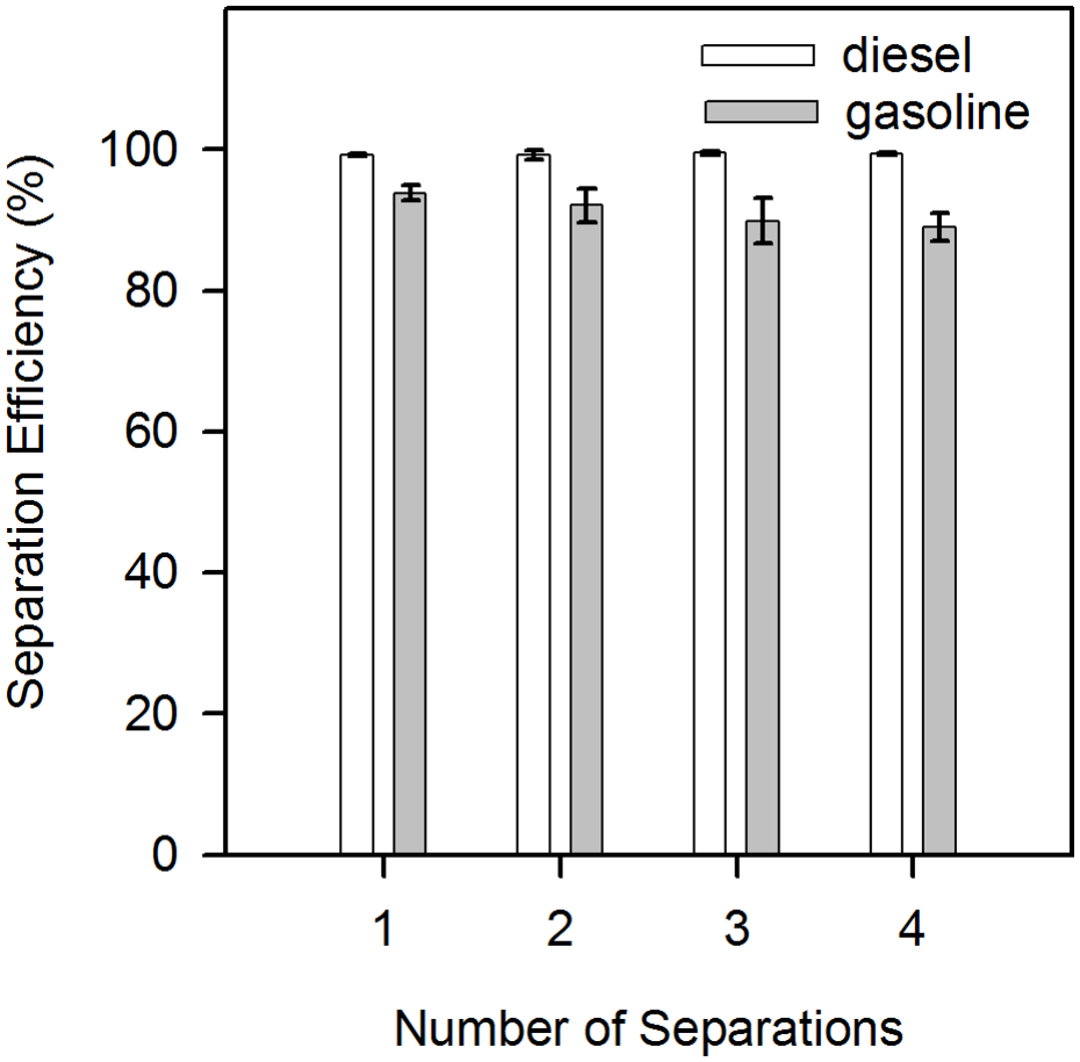
Relationship between separation efficiency of oil/water mixtures and the number of separations on a single sheet of silanized filter paper.

Based on the above study, further research on the oil/water separation efficiency of the hydrophobic filter paper was carried out. Herein, the separation efficiency was defined as the ratio of the weight of water after filtration to the weight of the original amount added [35]. As shown in Fig 6, both diesel and gasoline can be efficiently separated from water when mixed at a 1:1 ratio. What is more fascinating is that the modified filter paper can be reused, with little change in separation efficiency. This is in contrast to the wetting properties and morphological changes mentioned above, particularly in the case of diesel/water separation. In fact, the separation efficiency for diesel/water mixtures (1:1) and the reusability appeared to be even superior to that of gasoline-water mixtures. As depicted in Fig 6, after being reused for four times, the separation efficiency for a 1:1 mixture of diesel/water exceeded 97%, much higher than that of a gasoline-water mixture (89%).

**Fig 7:**
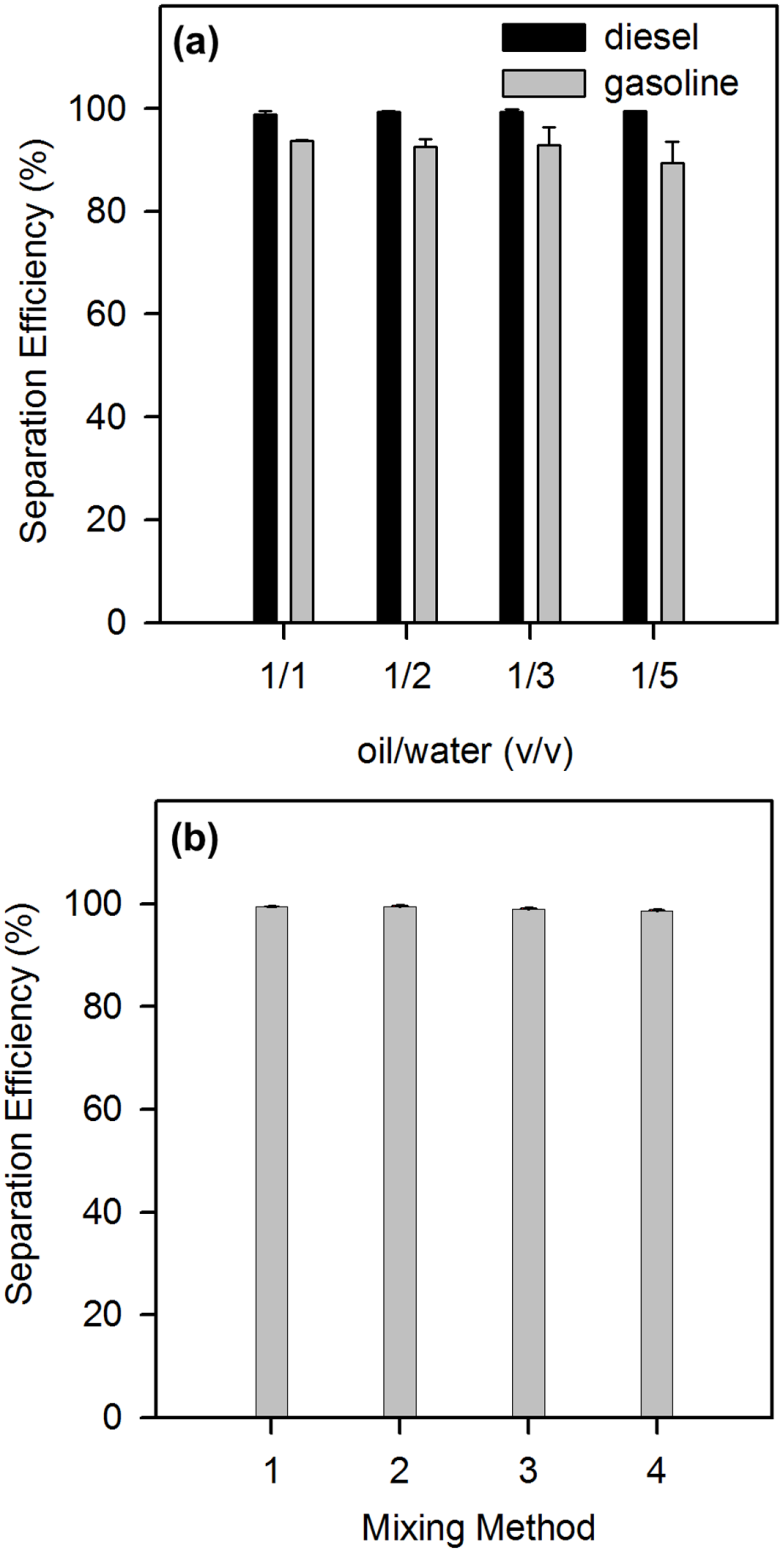
Separation efficiency of the modified filter paper for (a) different ratios of oil/water mixtures and (b) different preparation methods of the oil (diesel)/ water mixture. (1) Water and diesel oil were mixed without resting; (2) water and diesel oil were mixed, vibrated for 5 min, without resting; (3) water and diesel oil were mixed, vibrated for 5 min and rested for 2 days; (4) water and diesel oil were ultrasonically mixed for 5 min and rested for 2 days.

To determine if the separation efficiency is dependent on the initial composition of oil/water mixtures, different ratios (ranging from 1:5 to 1:1) of oil and water were mixed and tested for separation with the modified filter paper. The test was repeated in triplicate and the results are shown in Fig 7a. It is clear that for the case of diesel, the separation efficiency is independent of the tested diesel/water ratios. Compared to diesel, the separation efficiency of gasoline-water mixtures is lower (albeit still greater than 90%) and decreases when its percentage becomes lower in the mixture. We speculate that the differences could be related to the nature of the oil (different hydrocarbon chain lengths); further studies are warranted to understand this unique phenomenon.

Practically, domestic sewage or industrial wastewater that contains water and oil mixtures are not mixed by simple pouring one into the other, but rather the mixture usually undergoes mechanical or ultrasonic shaking and subsequent resting. This was simulated in a controlled experiment to test whether different mixing methods (e.g., four different agitation and resting combinations) affect the separation efficiency. The first method consisted of direct mixing of water and diesel in the modified filter set up (similar to that shown in Fig 3) for separation without resting time. In the second method, water and diesel were mixed and shaken for 5 min before separation. In the third and fourth method, a resting time of 2 days was given after mixing by 5-min shaking and ultra-sonication, respectively. As shown in Fig 7b, the mixing method had little effect on the separation efficiency; different mixing methods have similar separation efficiencies (the highest is 99.4%, the lowest 98.6%). When we took experimental errors into consideration, there is merely a subtle difference between them. That is to say, the as-prepared superhydrophobic filter paper possessed excellent separation ability and selectivity for oil/water mixtures despite the differences in mixing methods and compositions.

## Conclusions

Inspired by the growing need for treating industrial wastewater and recovering ecosystems, we have developed a simple method to modify conventional filter paper into a nanostructured, superhydrophobic material for efficient oil/water separation, based on our newly developed binary silanization reactions. Thus modified filter paper is stable to acidic and alkaline environments, as well as common organic solvents involved in environmental spills. In addition, the silanized filter paper can be reused multiple times and easily disposed of after use. Because of paper’s ease of accessibility, the modified filter paper promises to be a great candidate for future oil/water separation in industrial and environmental settings.

## Materials and Methods

Octadecyltrichlorosilane (OTS, ≥90%) and methyltrichlorosilane (MTS, 99%) were purchased from Sigma-Aldrich and used as received. Different types of filter paper (Whatman^™^, Grades 1, 3, 541) and regular printing paper (Georgia Pacific, Advantage^®^) were cut into appropriate size (circle, diameter ≈ 90 mm to fit a standard glass funnel after folding). The paper was then immersed into the OTS/MTS solution in n-hexane (Aladdin) at ambient temperature (25±2°C) for different periods of time (from 1 to 8 min). Subsequently, the paper was removed from the solution, heated and dried in an oven (GZX-9070 MBE, Shanghai Boxun) at 40°C for 5 min before any further characterization.

The surface morphology of the filter paper (unmodified, modified, and after separation experiments) was examined using scanning electron microscopes (Helios Nanolab 650 and Nova NanoSEM, FEI). The wetting properties of the paper were studied using a digital contact angle system (VCA Optima, AST). Water contact angles were measured 6 times on each sample, and at least three replicates were tested.

The oil/water separation experiment was conducted by folding the as-prepared filter paper into quarters (in half twice) to make a cone, which was then placed into a conical glass funnel on a ring stand. Mixtures of oil (diesel or gasoline, obtained from a local gas station) and water, mixed by using a spiral agitator (SK-1, Jintan, Jiangsu) or an ultrasonic cleaner (KQ-100DE, Kunshan, Jiangsu), were poured into the filter paper-covered funnel for separation. The weights of the water, before and after the separation, were measured using an analytical balance (CP224C, OHAUS) and these values were used for the determination of oil/water separation efficiency. For testing the stability of the filter paper, aqueous solutions of different pH values ranging from 1 to 13 (1, 3, 7, 10, 13) were prepared with concentrated HCl and NaOH, respectively. The modified filter paper was placed into 10 mL of each solution (fully immersed) for 12 hours; it was dried again at 40°C, before water contact angles were measured.

## References

[1] Xue Z, Wang S, Lin L, Chen L, Liu M, Feng L, et al A novel superhydrophilic and underwater superoleophobic hydrogel-coated mesh for oil/water separation. Adv Mater. 2011;23: 4270–4273. 22039595 10.1002/adma.201102616

[2] Wang L, Zhao Y, Tian Y, Jiang L. A general strategy for the separation of immiscible organic liquids by manipulating the surface tensions of nanofibrous membranes. Angew Chem Int Ed. 2015;54:14732–14737. 10.1002/anie.201506866 26492856

[3] Atlas RM. Microbial degradation of petroleum hydrocarbons: an environmental perspective. Microbiol Rev. 1981;45: 180–209. 7012571 10.1128/mr.45.1.180-209.1981 PMC281502

[4] Dai W, Kim SJ, Seong W-K, Kim SH, Lee K-R, Kim H-Y, et al Porous carbon nanoparticle networks with tunable absorbability. Sci Rep. 2013;3: 1–9. 10.1038/srep02524 PMC3755281 23982181

[5] Zhang M, Wang C, Wang S, Li J. Fabrication of superhydrophobic cotton textiles for water-oil separation based on drop-coating route. Carbohydr Polym. 2013;97: 59–64. 3 10.1016/j.carbpol.2012.08.118 23769517

[6] Zhu Q, Pan Q, Liu F. Facile removal and collection of oils from water surfaces through superhydrophobic and superoleophilic sponges. J Phys Chem C. 2011;115: 17464–17470.

[7] Chen P-C, Xu Z-K. Mineral-coated polymer membranes with superhydrophilicity and underwater superoleophobicity for effective oil/water separation. Sci Rep. 2013;3: 2776 10.1038/srep02776 24072204 PMC3784956

[8] Inagaki M, Kawahara A, Nishi Y, Iwashita N. Heavy oil sorption and recovery by using carbon fiber felts. Carbon 2002 40: 1487–1492.

[9] Venkateswara Rao A, Hegde ND, Hirashima H. Absorption and desorption of organic liquids in elastic superhydrophobic silica aerogels. J Colloid Interface Sci. 2007;305: 124–132. 17067617 10.1016/j.jcis.2006.09.025

[10] Zhang Y, Wei S, Liu F, Du Y, Liu S, Ji Y, et al Superhydrophobic nanoporous polymers as efficient adsorbents for organic compounds. Nano Today. 2009;4: 135–142.

[11] Radetić MM, Jocić DM, Jovančić PM, Petrović ZL, Thomas HF. Recycled wool-based nonwoven material as an oil sorbent. Environ Sci Technol. 2003;37: 1008–1012. 12666933 10.1021/es0201303

[12] Yetisen AK, Akram MS, Lowe CR. Paper-based microfluidic point-of-care diagnostic devices. Lab Chip. 2013;13: 2210–51. 10.1039/c3lc50169h 23652632

[13] Wang H, Fang J, Cheng T, Ding J, Qu L, Dai L, et al One-step coating of fluoro-containing silica nanoparticles for universal generation of surface superhydrophobicity. Chem Commun. 2008; 877–879. 10.1039/b714352d 18253534

[14] Arbatan T, Zhang L, Fang XY, Shen W. Cellulose nanofibers as binder for fabrication of superhydrophobic paper. Chem Eng J. 2012;210: 74–79.

[15] Li S, Zhang S, Wang X. Fabrication of superhydrophobic cellulose-based materials through a solution-immersion process. Langmuir. 2008;24: 5585–5590. 10.1021/la800157t 18426232

[16] Huang X, Wen X, Cheng J, Yang Z. Sticky superhydrophobic filter paper developed by dip-coating of fluorinated waterborne epoxy emulsion. Appl Surf Sci. 2012;258: 8739–8746.

[17] Li J, Wan H, Ye Y, Zhou H, Chen J. One-step process to fabrication of transparent superhydrophobic SiO_2_ paper. Appl Surf Sci. 2012;261: 470–472.

[18] Zhang M, Wang C, Wang S, Shi Y, Li J. Fabrication of coral-like superhydrophobic coating on filter paper for water-oil separation. Appl Surf Sci. 2012;261: 764–769.

[19] Ge B, Zhu X, Li Y, Men X, Li P, Zhang Z. The efficient separation of surfactant-stabilized water-in-oil emulsions with a superhydrophobic filter paper. Appl Phys A. 2015;121: 1291–1297.

[20] Du C, Wang J, Chen Z, Chen D. Durable superhydrophobic and superoleophilic filter paper for oil-water separation prepared by a colloidal deposition method. Appl Surf Sci. 2014;313: 304–310.

[21] Kong L, Wang Q, Xiong S, Wang Y. Turning low-cost filter papers to highly efficient membranes for oil/water separation by atomic-layer-deposition-enabled hydrophobization. Ind Eng Chem Res. 2014;53: 16516–16522.

[22] Fan J-B, Song Y, Wang S, Meng J, Yang G, Guo X, et al Directly coating hydrogel on filter paper for effective oil-water separation in highly acidic, alkaline, and salty environment. Adv Funct Mater. 2015; 25: 5368–5375.

[23] Li S, Zhang S, Wang X. Fabrication of superhydrophobic cellulose-based materials through a solution-immersion process. Langmuir. 2008;24:5585–5590. 10.1021/la800157t 18426232

[24] He Q, Ma C, Hu X, Chen H. Method for fabrication of paper-based microfluidic devices by alkylsilane self-assembling and UV/O_3_-patterning. Anal Chem. 2013;85: 1327–1331. 10.1021/ac303138x 23244032

[25] McGovern ME, Kallury KMR, Thompson M. Role of solvent on the silanization of glass with octadecyltrichlorosilane. Langmuir. 1994;10: 3607–3614.

[26] Wong JXH, Asanuma H, Yu H-Z. Simple and reproducible method of preparing transparent superhydrophobic glass. Thin Solid Films. 2012;522: 159–163.

[27] Wong JXH, Yu H-Z. Preparation of transparent superhydrophobic glass slides: Demonstration of surface chemistry characteristics. J Chem Educ. 2013;90: 1203–1206.

[28] GE Healthcare Life Sciences. Standard Grade. [Internet]. Available at: http://www.gelifesciences.com/webapp/wcs/stores/servlet/catalog/en/GELifeSciences-ca/products /AlternativeProductStructure_16160/

[29] Teisala H, Tuominen M, Kuusipalo J. Adhesion mechanism of water droplets on hierarchically rough superhydrophobic rose petal surface. J Nanomater. 2011;2011: 1–6.21808638

[30] Jin M, Feng X, Feng L, Sun T, Zhai J, Li T, et al Superhydrophobic aligned polystyrene nanotube films with high adhesive force. Adv Mater. 2005;17: 1977–1981.

[31] Guo Z-G, Liu W-M. Sticky superhydrophobic surface. Appl Phys Lett. 2007;90: 223111.

[32] Cassie ABD, Baxter S. Wettability of porous surfaces. Trans Faraday Soc. 1944;40: 546–551.

[33] Wang S, Li M, Lu Q. Filter paper with selective absorption and separation of liquids that differ in surface tension. ACS Appl Mater Interfaces. 2010;2: 677–683. 10.1021/am900704u 20356268

[34] Wenzel RN. Resistance of solid surfaces to wetting by water. J Ind Eng Chem. 1936;28: 988–994.

[35] Pan Q, Wang M, Wang H. Separating small amount of water and hydrophobic solvents by novel superhydrophobic copper meshes. Appl Surf Sci. 2008;254: 6002–6006.

